# High-Performance Mortar with Epoxy-Coated Lightweight Aggregates for Marine Structures

**DOI:** 10.3390/ma18184257

**Published:** 2025-09-11

**Authors:** Jin-Su Kim, Ho-Yeon Lee, Jang-Ho Jay Kim

**Affiliations:** School of Civil and Environmental Engineering, Yonsei University, 50, Yonsei-ro, Seodaemun-gu, Seoul 03722, Republic of Korea; kjinsu@yonsei.ac.kr (J.-S.K.); hoyeon9710@yonsei.ac.kr (H.-Y.L.)

**Keywords:** high-performance mortar, bottom ash, artificial lightweight fine aggregate, mechanical properties, chloride penetration resistance

## Abstract

Due to the global growth of the construction industry, the use of concrete has increased rapidly. Consequently, the depletion of natural aggregates, which are essential components of concrete, has emerged as a critical issue. Simultaneously, the construction of marine structures has recently increased due to population growth and climate change. This trend highlights the growing demand for durable and sustainable construction materials in aggressive environments. To address the depletion of natural aggregates, extensive research has focused on artificial lightweight aggregates produced from industrial waste. However, the high porosity and low compressive strength of artificial lightweight aggregates have limited their effectiveness in ensuring the performance of sustainable marine structures. In this study, a high-performance mortar (HPM) incorporating artificial lightweight fine aggregates (ALWFAs) was developed to address the depletion of natural aggregates and to serve as a protective layer material in marine environments. To enhance the physical properties of ALWFAs, the aggregates were coated with epoxy-TiO_2_ coatings applied to both their internal voids and external surfaces. The effectiveness of this enhancement was assessed by comparing the performance of mortars prepared with uncoated and coated ALWFAs. The HPM was evaluated for its porosity, compressive strength, split tensile strength, and chloride diffusion coefficient. The results showed that increases in the ALWFA replacement ratio led to a general reduction in performance. However, a comparison between uncoated and coated ALWFAs revealed that the coated aggregates led to improvements of up to 4.13%, 49.3%, 28.6%, and 52.0% in porosity, compressive strength, split tensile strength, and chloride diffusion coefficient, respectively. The study results are discussed in detail in the paper.

## 1. Introduction

Concrete generally comprises cement, water, and natural aggregates, which contribute to strength, workability, and cost efficiency. The cost efficiency of natural aggregates is attributed to their high volume fraction, typically accounting for approximately 70–80% of the total concrete volume [1]. Moreover, concrete offers a significant advantage due to its excellent durability, which protects steel rebar or other metallic construction materials from corroding [2,3]. These advantages have made concrete the most widely used material in construction industry.

Due to the global growth of the construction industry, the use of concrete has increased rapidly. Additionally, population growth and climate change have driven increasing demand for the construction of marine structures. Consequently, the depletion of natural aggregates (such as sand and gravel), which are essential components of concrete, has emerged as a critical issue [4,5]. Alternative aggregates have been continuously studied to address this depletion of natural aggregates. Nevertheless, the use of alternative aggregates in concrete often results in a significant reduction in strength and performance compared with natural aggregates. Chloride penetration through the pore structure of aggregates in concrete under marine conditions causes reinforcement corrosion, which significantly reduces the service life of marine structures. These structures are continuously exposed to high-chloride and high-humidity environments and are subjected to various aggressive actions from seawater, resulting in service lives that are often much shorter than their intended design life. Therefore, to address the depletion of natural aggregates and to enable application in marine environments, the development of improved alternative aggregates is essential. In addition, to ensure the sustainability of marine structures, it is essential to use concrete or mortar with high strength and durability [6,7].

In recent decades, researchers have continuously studied alternative aggregates to address the depletion of natural aggregates. In particular, significant attention has been given to the development of eco-friendly artificial lightweight aggregates utilizing low-cost industrial waste by-products. Bottom ash, an industrial by-product generated by coal power plants, has been widely used as a substitute for fine or coarse aggregates in concrete mixtures across various regions [8,9]. Studies on concrete incorporating artificial lightweight fine aggregates (ALWFAs) derived from bottom ash have focused on compressive strength, split tensile strength, flexural strength, durability, and microstructural properties. Hamada et al. investigated the mechanical properties of concrete according to the replacement ratio of ALWFAs and reported that compressive strength decreased by 20–50% and splitting tensile strength decreased by 12–58% as the replacement ratio increased [10,11,12,13,14,15]. Lynn BE and Bilir et al. examined the porosity and chloride diffusion coefficient in relation to the ALWFA replacement ratio and reported that porosity and chloride diffusion coefficient significantly increased when the replacement ratio exceeded 30% [16,17,18]. Previous studies on ALWFAs as substitutes for fine aggregates in concrete have shown that increasing the replacement ratio leads to deterioration in mechanical strength, porosity, and chloride resistance. This was attributed to the low strength and high porosity of the aggregate itself, as well as the formation of ettringite, which induces expansion and cracking [12]. While ALWFAs offer a potential solution to the depletion of natural aggregates, their performance in marine environments remains inadequate.

In this study, to address the depletion of natural aggregates and enable their application in marine structures, ALWFAs were enhanced by impregnating epoxy-TiO_2_ into the internal void and coating external surfaces under vacuum and pressure conditions [19]. The enhanced ALWFAs were incorporated into a high-performance mortar (HPM) through a cement mortar mix design. In the HPM specimens, silica sand was replaced with ALWFAs at replacement levels of 25%, 50%, 75%, and 100%. To compare the performance of uncoated and coated ALWFAs in HPM, compressive strength, split tensile strength, water absorption, porosity, and chloride diffusion coefficient were evaluated. The testing procedures are described in detail in the following sections.

## 2. Mix Proportion and Specimen Details

### 2.1. Artificial Lightweight Fine Aggregate

**Figure 1:**
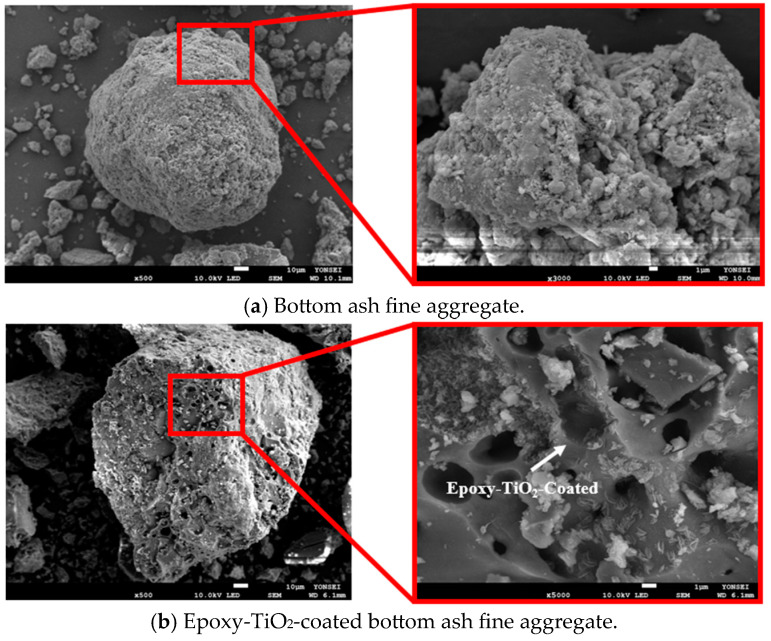
Image of artificial lightweight fine aggregate.

High-strength functional ALWFAs were fabricated by embedding epoxy into the internal pores (approximately 0.3–300 μm) of raw ALWFAs, while simultaneously applying a coating to their external surfaces. The epoxy-TiO_2_ coating was conducted from bisphenol-A epoxy resin with a polyamide curing agent, in which TiO_2_ nanoparticles were uniformly dispersed. The viscosity of the coating mixture was approximately 1000 cps at 25 °C. The coating was applied using a vacuum pump (SH–VDO–08NG, SH SCIENTIFIC, Sejong, South Korea) to fill the internal pores of the ALWFA, and the specimens were then immersed in epoxy to form an outer layer. The epoxy-TiO_2_ composite solution was left at a temperature of 20 °C for 60 min under atmospheric pressure (1 atm), followed by drying in ambient air for 8 h. For the vacuum pressure coating, the epoxy-TiO_2_ solution containing ALWFAs was placed in a vacuum oven and maintained under 600 Torr (≈0.79 atm) for 30 min. Afterwards, the ALWFAs were removed and dried at room temperature (20 °C) under atmospheric pressure for 8 h [19]. Finally, the coated ALWFAs were fully cured under laboratory conditions (20 ± 2 °C, atmospheric pressure) for 24 h. Figure 1 shows scanning electron microscope (SEM) images comparing normal ALWFAs and coated ALWFAs with the epoxy-TiO_2_ composite. As shown in Figure 1a, bottom ash particles agglomerated to form a pore structure in both the interior and exterior of the aggregate. In Figure 1b, it can be observed that both the internal and external surfaces of the ALWFAs were successfully reinforced with the epoxy-TiO_2_ composite. This internal impregnation with epoxy-TiO_2_ significantly improves both the porosity and the mechanical strength of the aggregate [19].

### 2.2. Substrate Materials

**Figure 2:**
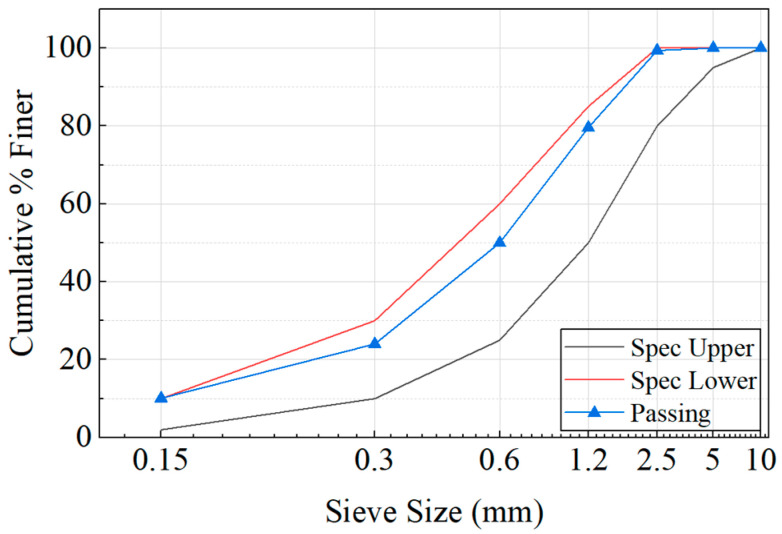
Particle size distribution of ALWFAs.

The binder used in the HPM consisted of type I ordinary Portland cement (OPC) and silica fume (SF) with unit weights of 3150 and 2200 kg/m^3^, respectively. The fillers used to mix the HPM were silica powder (SP), SS, and ALWFAs with unit weights of 2650, 2650, and 1080 kg/m^3^, respectively. The water absorption, porosity, and unit weight of the ALWFAs were measured as 19.1%, 52.2%, and 1080 kg/m^3^, respectively. For the coated ALWFAs, the unit weight was measured as 1700 kg/m^3^, and their water absorption was revealed to have decreased [19]. The particle size distribution and XRF results of the uncoated ALWFAs are shown in Figure 2 and Table 1, respectively. The main phases of the ALWFAs were identified as SiO_2_ and Al_2_O_3_, with proportions of 45.50% and 23.40%, respectively.

### 2.3. HPM Mixing Proportions and Curing

The high-performance mortar (HPM) was composed of water, cement, silica fume (SF), silica powder (SP), silica sand (SS), and artificial lightweight fine aggregates (ALWFAs). Cement and SF were mixed at a ratio of 20:3, and the water-to-binder ratio was fixed at 1:4. Due to the low water-to-binder ratio of HPM, which resulted in reduced workability, a high-performance superplasticizer (HPSP) was added at 1% of the binder weight.

As shown in Table 2, the control consisted of cement, SF, SP, and SS in standard without ALWFAs. The control served as a benchmark to evaluate the effect of various replacement ratios of ALWFAs. To evaluate the performance of ALWFAs in HPM, the SS of the control was replaced with ALWFAs at 25%, 50%, 75%, and 100%, which corresponded to volume replacement ratios of 10.7%, 21.5%, 32.5%, and 43.0%, respectively. Furthermore, both uncoated and coated ALWFAs were used at the equivalent replacement ratios to allow for performance comparison. The ALWFAs used in this study were manufactured from bottom ash generated by a coal power plant. The same bottom-ash-based ALWFAs were consistently used in both the uncoated (NEC) and epoxy-TiO_2_-coated (EC) conditions.

Cubic specimens (50 × 50 × 50 mm) and cylindrical specimens (Φ100 × 200 mm and Φ100 × 50 mm) were prepared for compressive strength, split tensile strength, absorption/void ratio, mercury intrusion porosimetry (MIP), and chloride diffusion tests according to ASTM C109, ASTM C496, ASTM C642, ASTM D4404, and NT-BUILD 492, respectively [20,21,22,23,24].

Marine structures are generally difficult to cast in the field. Therefore, precast concrete is commonly used. Precast concrete is cured under high temperature and high humidity conditions. In addition, based on the characteristics of HPM, more than 90% of the hydration reaction occurred when cured at a temperature above 90 °C for more than 2 days [25,26,27]. After demolding, the specimens were cured in an oven with a temperature of 90 °C under 95% relative humidity for 2 days.

## 3. HPM Evaluation Procedures

### 3.1. HPM Porosity

The porosity and water absorption of HPM were evaluated according to ASTM C642. The specimens were dried in an oven at 110 ± 5 °C until a constant mass was reached, and the mass of the oven-dried sample in air (A) was measured using a precision balance. The dried specimens were immersed in water at room temperature for 48 h to ensure saturation. After wiping the surface with a damp cloth, the mass of the surface-dried sample after immersion (B) was measured. The mass of the surface-dried sample after immersion and boiling (C) was measured after boiling the specimens in water for 5 h and cooling to room temperature. Finally, the apparent mass in water after immersion and boiling (D) was measured by suspending the specimen in water. Based on these measurements, the porosity and water absorption were calculated using Equations (1) and (2) [22].

(1) $$Water\;Absorption=\frac{B-A}{A}\times 100(\%)$$

(2) $$Porosity=\frac{C-A}{C-D}\times 100(\%)$$

where A is the mass of the oven-dried sample in air (g), B is the mass of the surface-dried sample in air after immersion (g), C is the mass of the surface-dried sample in air after immersion and boiling (g), and D is the apparent mass of the sample in water after immersion and boiling (g).

The porosity and pore diameter of HPM were evaluated using the mercury intrusion porosimetry according to ASTM D4404. After the compressive strength test, the specimens were crushed to a size of 2–5 mm to facilitate mercury intrusion. The crushed samples were immersed in the alcohol-based solvent (i.e., isopropanol) for 1 h to remove the remaining water, and then oven-dried at 60 °C for 24 h to remove residual moisture. After drying, the samples were weighed using an analytical balance with a precision of 0.0001 g. Mercury intrusion data obtained under controlled pressure were used to calculate the total porosity and average pore diameter using Equations (3) and (4) [23].

(3) $$Porosity\left(\%\right)=\frac{V_{Hg}}{V_{s}}\times 100$$

where $V_{Hg}$ is the total volume of mercury intruded (cm^3^), and $V_{s}$ is the sample volume (cm^3^).

(4) $$d=\frac{-4\gamma cos\theta }{P}$$

where d is the pore size (μm), γ is the surface tension of mercury (N/m), θ is the contact angle of mercury (∘), and P is the applied pressure (Pa).

### 3.2. Compressive Strength Test

A universal testing machine with a maximum capacity of 1000 kN, with an accuracy of 500 N, was used for the compressive strength test. Cubic specimens (50 × 50 × 50 mm) were prepared according to the ASTM C 109 standards; these were demolded after 24 h and cured in an oven at 90 °C with 95% relative humidity for 48 h. The end surfaces of the specimens were aligned in the axial direction, and load control was applied at a rate of 0.6 ± 0.2 MPa/s [20].

### 3.3. Split Tensile Strength Test

The split tensile strength test was conducted on cylindrical specimens (Φ100 × 200 mm) by ASTM C496. All specimens were demolded after 24 h and cured in an oven at 90 °C with 95% relative humidity for 48 h, followed by storage under ambient conditions until the day of testing. The curing process was consistent with that used for the compressive strength evaluation. Tests were performed using a universal testing machine (SGB-201-1A, SHINGANG PRECISIONIND CO. LTD., Seoul, South Korea) with a maximum capacity of 1000 kN. The load was applied uniformly along the vertical diameter of each specimen under a load control rate of 1.0 ± 0.2 MPa/min [21].

### 3.4. Chloride Migration Test

A vacuum was used to saturate Φ100 × 50 mm cylindrical HPM specimens in a saturated Ca(OH)_2_ solution for 1 day to fill the internal pores. NaOH (0.3 Mol) solution was placed in the anode compartment, and NaCl (10%) solution was placed in the cathode compartment, with an application of 60 V voltage. The specimen was split along its vertical axis using the split tensile strength test setup. The fractured surface was sprayed with AgNO_3_ (0.1 Mol) solution, and the chloride penetration depth was measured as the boundary between white and brown zones. The initial current value, temperature, and chloride penetration depth were measured to calculate a chloride diffusion coefficient using Equation (5) [24].

(5) $$D_{nssm}=\frac{0.0239\left(273+T\right)L}{\left(U-2\right)t}(X_{d}-0.0238\sqrt{\frac{\left(273+T\right)LX_{d}}{U-2}})$$

where $D_{nssm}$ is a non-steady-state migration coefficient (m^2^/s); U is the absolute value of the applied voltage (V); T is the average value of the initial and final temperatures in the anolyte solution (°C); L is the thickness of the specimen (mm); $X_{d}$ is the average value of the penetration depth (mm); t is the test duration (h).

## 4. Results and Discussions

### 4.1. HPM Porosity Result

**Figure 3:**
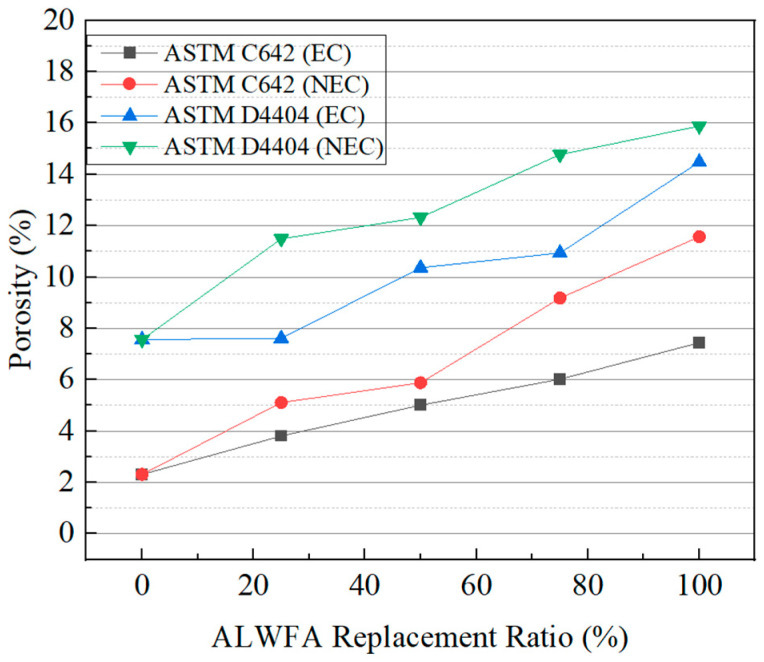
Comparison of porosity between ASTM C642 and ASTM D4404.

The absorption and porosity test results of the HPM specimens, conducted according to ASTM C642 and ASTM D4404, are shown in Table 3 and Figure 3. According to the ASTM C642 absorption results, the control specimen (ALWFA 0%) showed the lowest absorption rate of 1.05%. The absorption rate increased gradually with higher replacement ratios of ALWFAs. The absorption rates of the NEC using uncoated ALWFA replacement ratios of 25%, 50%, 75%, and 100% were 2.43%, 2.84%, 4.73%, and 6.17%, respectively. For the EC using coated ALWFAs, the corresponding absorption rates were 1.84%, 2.52%, 3.19%, and 4.08%. The EC specimens showed reductions in absorption rate ranging from 0.32% to 2.09%, at equivalent replacement ratios of ALWFAs.

According to the ASTM C642 porosity test results of the HPM specimens, the control specimen (ALWFA 0%) was measured to have the lowest porosity of 2.31%. The porosity increased with higher replacement ratios of ALWFAs. The porosity values of the NEC using uncoated ALWFA replacement ratios of 25%, 50%, 75%, and 100% were 5.11%, 5.88%, 9.18%, and 11.57%, respectively. For the EC using coated ALWFAs, the corresponding porosity values were 3.81%, 5.01%, 6.02%, and 7.44%, respectively. The EC specimens showed reductions in porosity rate ranging from 0.87% to 4.13%, at equivalent replacement ratios of ALWFAs.

According to the ASTM D4404 test results of the HPM specimens, the average pore diameter ranged from 228 to 498 nm, and no consistent trend was observed with respect to the replacement ratios. Because the MIP is sensitive to the condition of the mortar samples and involves pressure injection, no trend in the pore structure was observed. In contrast, the cumulative pore volume and porosity results exhibited consistent trends. As a result, the porosity results based on ASTM D4404 showed that the control specimen (ALWFA 0%) was measured to have the lowest porosity of 7.56%, and the porosity generally increased with higher replacement ratios of ALWFAs. The porosity values of the NEC using uncoated ALWFA at replacement ratios of 25%, 50%, 75%, and 100% were 11.49%, 12.32%, 14.77%, and 15.88%, respectively. For the EC using coated ALWFAs, the corresponding porosity values were 7.61%, 10.36%, 10.94%, and 14.48%, respectively. The EC specimens showed reductions in porosity ranging from 1.40% to 3.88%, at equivalent ALWFA replacement ratio. Overall, as the replacement ratios of ALWFAs increased, the absorption and porosity of the HPM specimens showed an increasing trend. This trend showed the inherent porosity of ALWFAs [10]. In contrast, a comparison between uncoated and coated ALWFAs showed that the internal pores of the ALWFAs were filled with epoxy-TiO_2_, resulting in lesser degradation compared with NEC in both absorption and porosity [19]. Furthermore, the porosity of HPM measured according to ASTM C642 and ASTM D4404 differed by a factor of 2 to 3, which can be attributed to the testing method of ASTM D4404. This method involves injecting high-pressure mercury into crushed specimens, allowing for the measurement of connected fine pores within the internal structure [28]. ASTM C642 measures open porosity through which water penetrates, based on changes in mass due to water absorption. On the other hand, ASTM D4404 measures open porosity, porosity not completely saturated with water, and smaller capillary porosity. Therefore, ASTM D4404 typically measures smaller pore structures associated with ion transport by providing higher porosity values. Chloride diffusion in marine structures is mainly affected by porosity through which water can penetrate. Therefore, ASTM C642 directly represents porosity contributing to chloride penetration under field conditions. On the other hand, ASTM D4404 provides complementary information on the microscopic pore structures that affect long-term diffusivity.

### 4.2. Compressive Strength Result

**Figure 4:**
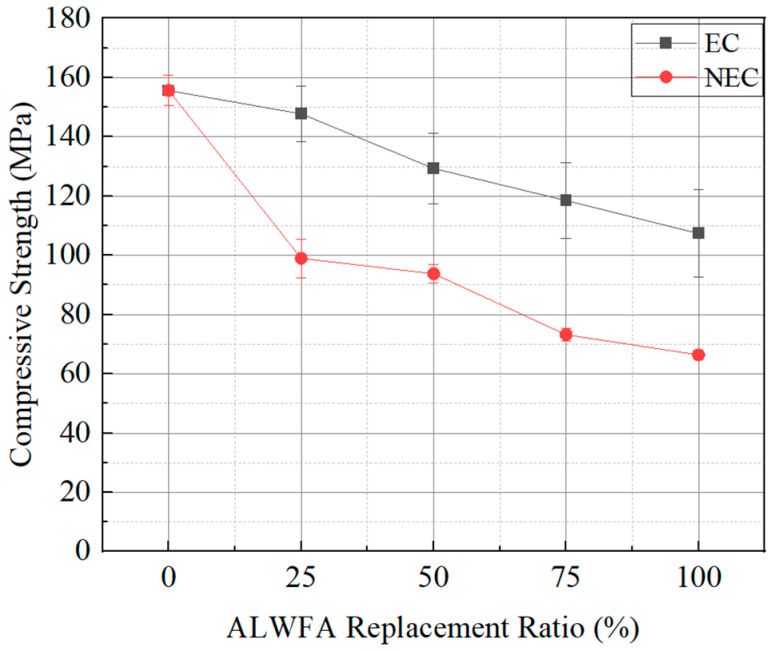
Compressive strength results of the HPM with various ALWFA replacement ratios.

The compressive strength test results of the HPM specimens are shown in Table 4 and Figure 4. The control specimen (ALWFA 0%) showed the highest compressive strength of 155.62 MPa, while the compressive strength of the specimens generally decreased as the replacement ratios of ALWFAs increased. The compressive strengths of the NEC using uncoated ALWFAs with replacement ratios of 25%, 50%, 75%, and 100% were 98.97, 93.76, 73.17, and 66.37 MPa, respectively. The NEC 100% specimen showed a compressive strength of 66.37 MPa, which did not satisfy the required compressive strength of 80 MPa for HPM. This result shows that a replacement of 100% with uncoated ALWFAs is unlikely to be feasible for high-performance structural applications. In comparison, the compressive strengths of the EC using coated ALWFA were 147.74, 129.31, 118.48, and 107.36 MPa at the corresponding replacement ratios. Compared to the control specimen, the compressive strength of the NEC using uncoated ALWFA with replacement ratios of 25%, 50%, 75%, and 100% decreased by 36.4%, 39.8%, 53.0%, and 57.4%, respectively. For the EC using coated ALWFAs, the compressive strength decreased by 5.1%, 16.9%, 23.9%, and 31.0% at equivalent replacement ratios of ALWFAs, respectively. Similar to previous studies on concrete incorporating ALWFAs, an increase in the ALWFA replacement ratio led to a significant reduction in compressive strength. Kim et al. (2011) reported that when the ALWFA replacement ratio exceeded 30%, compressive strength sharply decreased [29]. Bogas et al. (2013) reported that the reduction in strength of concrete using ALWFAs is mainly attributed to its high porosity and the weak interfacial transition zone [30]. This study supports these conclusions, as the NEC using uncoated ALWFAs showed a sharp decrease in strength beyond the replacement ratio of 50%. In particular, the replacement of SS with uncoated ALWFAs in the NEC resulted in a sharper reduction in strength, whereas the use of coated ALWFAs in the EC mitigated the rate of strength degradation. Compared to the NEC, the EC revealed increases in compressive strength ranging from 35.55 to 48.77 MPa at equivalent replacement ratios of ALWFAs. These results indicate that the epoxy-TiO_2_ coating effectively enhances the aggregate strength and reduces porosity [19]. In addition, the ACI 318 and Eurocode 2 standards recommend compressive strengths of 30–35 MPa and 30–37 MPa, respectively, depending on the marine environmental classification of structures. The compressive strength in this study targeted values exceeding 80 MPa, which is the standard for HPM. Thus, the results are considered to meet the requirements of both the ACI and EN codes [31,32].

### 4.3. Split Tensile Strength Result

**Figure 5:**
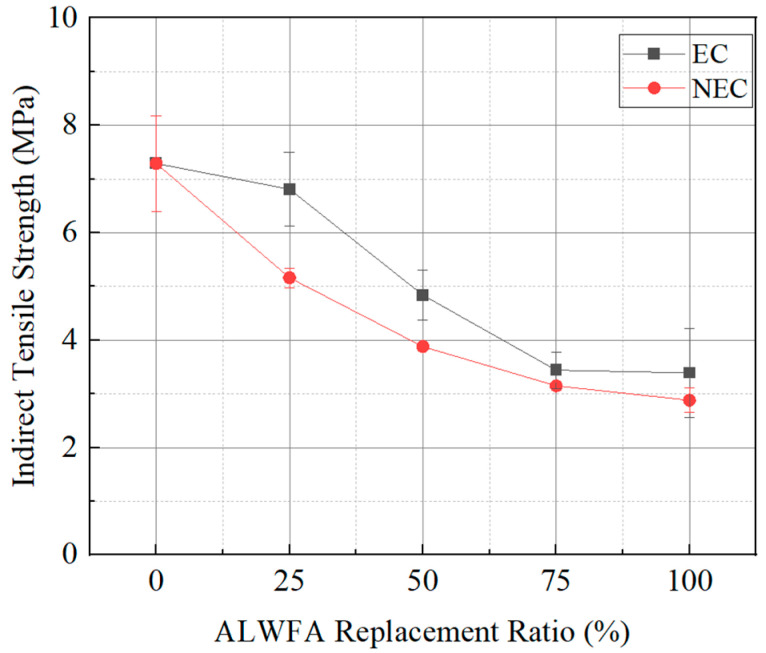
Split tensile strength results of the HPM with various ALWFA replacement ratio.

The split tensile strength test results of the HPM specimens are shown in Table 5 and Figure 5. The control specimen (ALWFA 0%) was measured as having the highest split tensile strength at 7.29 MPa, while the split tensile strength generally decreased as the replacement ratios of ALWFAs increased. The split tensile strengths of the NEC using uncoated ALWFAs at replacement ratios of 25%, 50%, 75%, and 100% were 5.16, 3.88, 3.15, and 2.88 MPa, respectively. In comparison, the corresponding strengths for the EC using coated ALWFA were 6.81, 4.84, 3.44, and 3.39 MPa. Compared to the control specimen, the split tensile strengths of the NEC using uncoated ALWFA replacement ratios of 25%, 50%, 75%, and 100% decreased by 29.2%, 46.8%, 56.8%, and 60.5%, respectively. For the EC, the decreases were 6.6%, 33.6%, 52.8%, and 53.5% at the equivalent replacement ratios of ALWFAs. Compared to the NEC, the EC revealed increases in split tensile strength ranging from 0.29 to 1.65 MPa at equivalent replacement ratios of ALWFAs [19]. The increased tensile strength can delay the development of microcracks and improve crack resistance. Since cracks are primary route for chloride penetration, the improved crack resistance can reduce chloride ingress in chloride-rich marine environments.

The decreasing trend in split tensile strength with increasing ALWFA replacement ratios is consistent with the observations reported in studies on lightweight aggregate concrete. Agrawal et al. (2021) reported that replacing ALFWAs led to a reduction in splitting tensile strength, attributed to the reduced bonding capacity resulting from the aggregate of high porosity and weak interfacial transition zone [33]. Similarly, Abed et al. (2022) reported 30–50% reduction in tensile strength when the ALWFA content increased from 25% to 75% [34]. The epoxy-TiO_2_ coating reduces porosity and interfacial micropores, improving compressive stress transfer and delaying the occurrence of microcracks. On the other hand, split tensile behavior is dominated by the initiation of cracks along the weakest interfaces. Despite the decrease in micropores, the increase in tensile strength is limited due to residual defects. In structural applications, mortar contributes to compressive strength, while reinforcement provides tensile strength. The tensile strength of mortar is limited in practical marine structures. Therefore, the measured split tensile strength is not regarded as a parameter for marine structural design, but rather as an indicator to confirm the mechanical improvement trend imparted by the coated ALWFA.

### 4.4. Chloride Penetration Resistance

**Figure 6:**
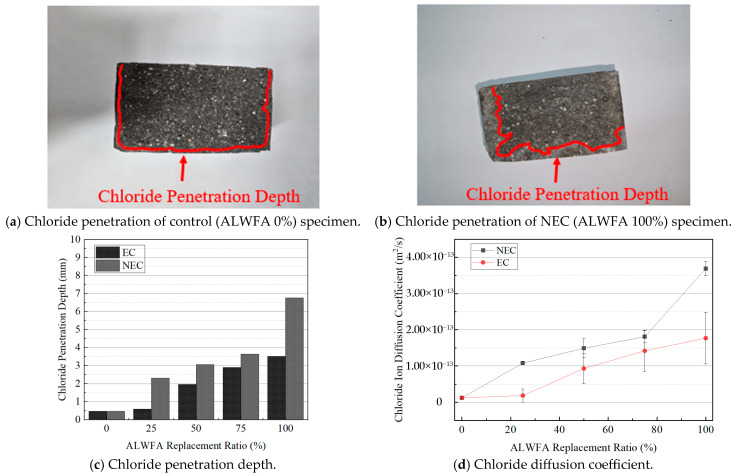
Chloride penetration resistance results for various ALWFA replacement ratios.

To evaluate the chloride penetration resistance, the NT-BUILD 492 test was conducted. The chloride penetration depth results are shown in Table 6 and Figure 6a–c. According to NT-BUILD 492, the control specimen (ALWFA 0%) was measured as having the lowest penetration depth at 0.455 mm. As the replacement ratios of ALWFAs increased, the penetration depth of the specimens increased. The penetration depths for the NEC using uncoated ALWFAs at replacement ratios of 25%, 50%, 75%, and 100% were 2.310, 3.060, 3.641, and 6.773 mm, respectively. In contrast, the EC using coated ALWFAs showed corresponding values of 0.585, 1.965, 2.893, and 3.517 mm. The chloride penetration depths of the EC specimens were reduced by 0.748–3.256 mm.

The chloride diffusion coefficient of the HPM specimens was calculated according to NT-BUILD 492 using Equation (5). The results are shown in Table 6 and Figure 6d. The control specimen was measured as having the lowest chloride diffusion coefficient at 1.26 × 10^−14^ m^2^/s. As the replacement ratios of ALWFA increased, the chloride diffusion coefficient of the specimens increased. The chloride diffusion coefficients of the NEC using uncoated ALWFAs at replacement ratios of 25%, 50%, 75%, and 100% were 1.08 × 10^−13^, 1.49 × 10^−13^, 1.81 × 10^−13^, and 3.69 × 10^−13^ m^2^/s, respectively. In comparison, the EC using coated ALWFAs showed corresponding values of 1.84 × 10^−14^, 9.30 × 10^−14^, 1.42 × 10^−13^, and 1.77 × 10^−13^ m^2^/s. The observed increase in both chloride penetration depth and diffusion coefficient is revealed to the high porosity of the ALWFAs [30]. This is consistent with the porosity test results in this study, which showed that the porosity of HPM increased with higher ALWFA replacement ratios. In this study, the coated ALWFAs demonstrated reduced porosity in the EC compared to the NEC, owing to the internal and external epoxy-TiO_2_ coatings. Furthermore, the chloride diffusion coefficients of the EC were reduced by 3.90 × 10^−13^ to 1.92 × 10^−13^ m^2^/s compared to the NEC at the equivalent replacement ratios of ALFWA. The fib Model Code 2010 provides exposure classes (XC, XD, XS) for concrete to specify durability requirements. The fib Model Code 2010 (based on the DuraCrete project) recommends reference diffusion coefficients of approximately 6 × 10^−12^ m^2^/s or less for XS3 of highest exposure condition to achieve a 50-year service life [35].

In this study, the HPM for marine structures set a compressive strength of 80 MPa and a chloride diffusion coefficient less than 1.00 × 10^−12^ m^2^/s. The HPM specimens using uncoated ALWFA (NEC) satisfied the compressive strength requirement at replacement ratios of 0–50%. However, at replacement ratios of 75–100%, the compressive strength fell below the required design. In contrast, all specimens satisfied the chloride diffusion coefficient criterion, indicating excellent resistance to chloride ingress. The HPM using coated ALWFAs (EC) satisfied both the compressive strength and chloride diffusion coefficient requirements at all replacement ratios of 0–100%. These results demonstrate that strengthening ALWFAs with epoxy-TiO_2_ effectively improves its performance, thereby enabling the development of environmentally friendly HPM that issuitable for marine environments.

### 4.5. Chloride Penetration Resistance of ACI Life 365 Standard

To evaluate the chloride penetration resistance of HPM, chloride concentrations obtained for the target structure under marine environmental conditions were analyzed. The prediction of chloride concentrations of a marine concrete structure can be conducted using a program based on the ACI Life 365 durability design criteria from the US. In this study, a structure with a column size of 600 mm × 600 mm × 10,000 mm is used. The boundary conditions of the ACI Life 365 program are shown in Table 7. Chloride penetration resistance is evaluated until the chloride concentration reaches a critical value of typically 1.2 to 2.5 kg/m^3^. The critical chloride concentration was set at 1.2 kg/m^3^ for a conservative evaluation. Furthermore, the highest surface chloride concentration was set at 18 kg/m^3^ in the splash zone, which is considered the highest among the conditions considered. Other assumptions include an age factor of 0.4, a surface chloride build-up time of 10 years, and a cover thickness of 30 mm.

**Figure 7:**
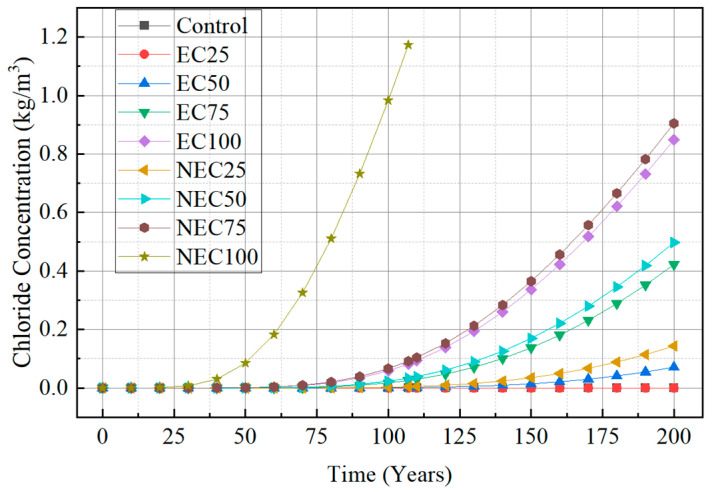
Chloride penetration resistance of the HPM with various ALWFA replacement ratios.

The chloride concentrations of the HPM specimens are shown in Figure 7. The control specimen (ALWFA 0%) was calculated as having the lowest chloride concentration of 0 kg/m^3^ over 100 years, while the chloride concentration generally increased as the replacement ratios of ALWFAs increased. The chloride concentration of the NEC using uncoated ALWFAs at replacement ratios of 25%, 50%, 75%, and 100% were 0.0027, 0.0228, 0.0662, and 0.9833 kg/m^3^, respectively. In comparison, the corresponding concentration for the EC using coated ALWFAs were 0, 0.0008, 0.0171 and 0.0591 kg/m^3^, respectively. The control specimen showed the lowest chloride concentration of 0 over 200 years. The chloride concentration of the NEC using uncoated ALWFAs at replacement ratios of 25%, 50%, and 75% were 0.0027, 0.0228, and 0.0662 kg/m^3^, respectively, and the NEC 100% specimen was calculated to fail after 107 years of chloride penetration resistance. In comparison, the corresponding concentration for the EC using coated ALWFAs were 0, 0.0708, 0.4218 and 0.8489 kg/m^3^, respectively. The control specimen showed the longest chloride penetration resistance life, and as the ALWFA replacement ratio increased, chloride penetration resistance decreased. Both the chloride concentration and chloride penetration resistance of the EC specimens with the equivalent ALWFA replacement ratio improved, and only the NEC 100% specimen failed after exceeding the critical chloride concentration of 1.2 kg/m^3^ within 200 years.

## 5. Conclusions

In this study, ALWFAs using bottom ash were coated with epoxy-TiO_2_ for application to marine structures. In addition, SS was partially replaced with ALWFAs; porosity, compressive strength, split tensile strength, and chloride diffusion coefficient tests of HPM using uncoated ALWFAs and coated ALWFAs were performed.

1. The porosity of HPM specimens according to ASTM C642 and ASTM D4404 standard showed a tendency to gradually increase as the replacement rate of ALWFAs increased. In the ASTM C642 standard, the porosity of EC was 0.87–4.13% lower than that of NEC at the equivalent replacement ratios of ALWFAs, and in the ASTM D4404 standard, the porosity of EC measured 1.40–3.88% lower than that of NEC.
2. The compressive strength of the HPM specimen gradually decreased as the replacement ratios of ALWFAs increased. The compressive strength of EC compared to NEC increased by 35.55–48.77 MPa depending on the equivalent replacement ratio of ALWFA. This suggests that the TiO_2_-epoxy coating effectively improves aggregate strength and reduces porosity. split tensile strength showed the same mechanism as compressive strength. The split tensile strength of EC improved by 0.29–1.65 MPa depending on the replacement ratio of the same ALWFA.
3. The chloride penetration depth and chloride diffusion coefficient of HPM decreased as the ALWFA replacement ratios increased. The chloride penetration depth of EC decreased by 0.748–3.256 mm and the chloride diffusion coefficient decreased by 3.90 × 10^−14^–1.92 × 10^−13^ m^2^/s compared to NEC at the equivalent ALWFA replacement ratio. This is attributed to the reduced porosity of the coated ALWFAs used in this study, as the interior and exterior coatings of EC compared to NEC contributed to a lower chloride diffusion coefficient. However, for the EC 100% specimen, the rapid reduction in performance compared with SS indicates that caution should be exercised in its application to marine structures.
4. The control specimen showed the longest chloride penetration resistance life, and as the ALWFA replacement ratio increased, the chloride penetration resistance life decreased. The chloride concentration and chloride penetration resistance life of the NEC and EC specimens with the equivalent ALWFA replacement ratio were improved, and only the NEC 100% specimen failed after exceeding the critical chloride concentration within 200 years.

## 6. Further Studies

Since the study has already been completed, data on the chemical composition and epoxy absorption of ALWFA are not available at this time. Therefore, in future study, more quantitative data from additional mixed epoxy ratios and viscosities using various epoxy types are required. In addition, the durability behavior with respect to chloride penetration resistance and carbonation and freeze–thaw tests will be quantified. From the reduced porosity and water absorption data obtained from this study, enhanced carbonation resistance and freeze–thaw durability can be expected. However, in the absence of the data, prediction of the long-term performance of ALWFA mortars in CO_2_-rich or freeze–thaw environments would be difficult and must be reconducted in future work.

## Figures and Tables

**Table 1:** Chemical compositions of substrate materials used in HMP.

Material	SiO_2_	Fe_2_O_3_	Al_2_O_3_	TiO_2_	CaO	MgO	Na_2_O	P_2_O_5_	K_2_O
OPC	18.78	2.76	4.72	0.31	65.97	3.14	-	0.19	-
SF	96.40	0.47	0.14	-	0.06	0.35	0.34	0.02	0.89
SP	99.00	0.06	0.30	0.06	0.02	0.02	0.05	-	0.02
SS	99.90	-	0.03	0.05	0.003	0.004	0.003	-	0.002
ALWFA	45.50	5.15	23.40	-	0.99	0.42	0.43	-	0.62

**Table 2:** HPM with various amounts of ALWFAs.

Sample	Water (kg/m^3^)	Cement (kg/m^3^)	SF (kg/m^3^)	SP (kg/m^3^)	SS (kg/m^3^)	ALWFA (kg/m^3^)	HPSP (kg/m^3^)
Control	178.46	713.86	178.46	178.46	915.20	-	8.20
NEC 25%					686.40	115.00	
NEC 50%					457.60	232.00	
NEC 75%					228.80	350.00	
NEC 100%					-	465.00	
EC 25%					686.40	185.75	
EC 50%					457.60	371.50	
EC 75%					228.80	557.25	
EC 100%					-	743.00	

**Table 3:** Porosity test results of ALWFA replacement ratio.

ALWFA Replacement Ratio (%)	ASTM C642				ASTM D4404			
	Absorption Rate (%)		Porosity (%)		Average Pore Diameter (nm)		Porosity (%)	
	NEC	EC	NEC	EC	NEC	EC	NEC	EC
0	1.05		2.31		498		7.56	
25	2.43	1.84	5.11	3.81	242	271	11.49	7.61
50	2.84	2.52	5.88	5.01	271	242	12.32	10.36
75	4.73	3.19	9.18	6.02	228	269	14.77	10.94
100	6.17	4.08	11.57	7.44	400	182	15.88	14.48

**Table 4:** Compressive strength results of ALWFA replacement ratio.

ALWFA Replacement Ratio (%)	Compressive Strength (MPa)		Standard Deviation		(σ_2_–σ_1_)
	NEC (σ_1_)	EC (σ_2_)	NEC	EC	
0	155.62		5.18		-
25	98.97	147.74	6.53	9.29	48.77
50	93.76	129.31	3.21	6.38	35.55
75	73.17	118.48	2.04	16.11	45.31
100	66.37	107.36	1.59	5.27	40.99

**Table 5:** Split tensile strength results of ALWFA replacement ratio.

ALWFA Replacement Ratio (%)	Split Tensile Strength (MPa)		Standard Deviation		(σ_2_–σ_1_)
	NEC (σ_1_)	EC (σ_2_)	NEC (σ_1_)	EC (σ_2_)	
0	7.29		1.22		-
25	5.16	6.81	0.18	0.47	1.65
50	3.88	4.84	0.03	0.46	0.96
75	3.15	3.44	0.08	0.34	0.29
100	2.88	3.39	0.23	0.83	0.51

**Table 6:** Chloride ion diffusion coefficient results of ALWFA replacement ratio.

ALWFA Replacement Ratio (%)	Chloride Penetration Depth (mm)		Standard Deviation		(X_D1_–X_D2_)
	NEC (X_D1_)	EC (X_D2_)	NEC	EC	
0	0.455		0.044		-
25	2.310	0.585	0.312	0.089	1.725
50	3.060	1.965	0.779	0.473	1.095
75	3.641	2.893	1.031	0.298	0.748
100	6.773	3.517	1.200	0.347	3.256
**ALWFA** **Replacement Ratio (%)**	**Chloride Ion Diffusion Coefficient (m^2^/s)**		**Standard Deviation**		**(D_1_–D_2_)**
	**NEC (D_1_)**	**EC (D_2_)**	**NEC**	**EC**	
0	1.26 × 10^−14^		2.00 × 10^−15^		-
25	1.08 × 10^−13^	1.84 × 10^−14^	1.82 × 10^−14^	4.22 × 10^−15^	8.96 × 10^−14^
50	1.49 × 10^−13^	9.30 × 10^−14^	4.19 × 10^−14^	2.59 × 10^−14^	5.60 × 10^−14^
75	1.81 × 10^−13^	1.42 × 10^−13^	5.67 × 10^−14^	1.67 × 10^−14^	3.90 × 10^−14^
100	3.69 × 10^−13^	1.77 × 10^−13^	7.13 × 10^−14^	1.96 × 10^−14^	1.92 × 10^−13^

**Table 7:** The boundary conditions used in the ACI Life 365 program.

Boundary Condition	Value
Critical Chloride Concentration	1.2 kg/m^3^
Surface Chloride Concentration	18 kg/m^3^
Age Factor	0.4
Surface Chloride Build-up Time	10 years
Propagation Period	6 years
Cover thickness	30 mm

## Data Availability

The original contributions presented in this study are included in the article. Further inquiries can be directed to the corresponding author.
